# Supplementary material for: ATR-FTIR Spectroscopy Combined with Multivariate Analysis Successfully Discriminates Raw Doughs and Baked 3D-Printed Snacks Enriched with Edible Insect Powder
Source: Foods. 2021 Aug 5;10(8):1806. doi: 10.3390/foods10081806 (PMC8394341; doi:10.3390/foods10081806)
Supplement: Supplementary file 1 [file foods-10-01806-s001.zip › foods-1301030-supplementary.pdf]

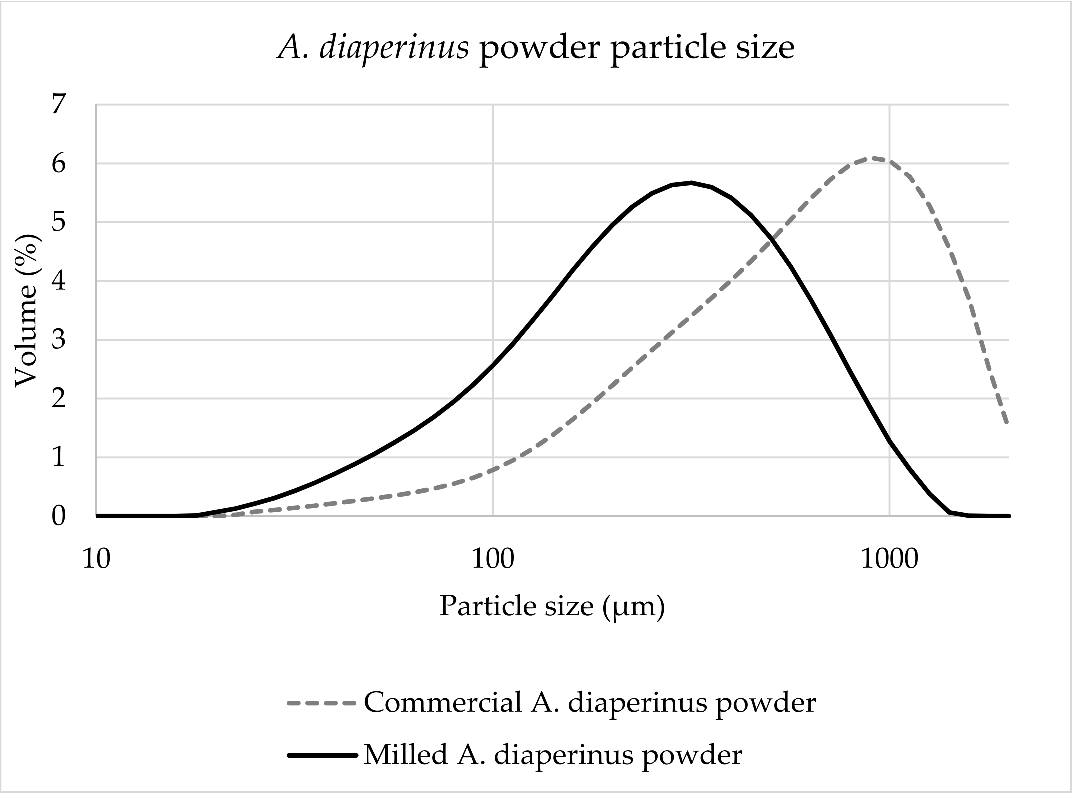

Figure S1. Particle size distribution of commercial and milled *A. diaperinus* powder.

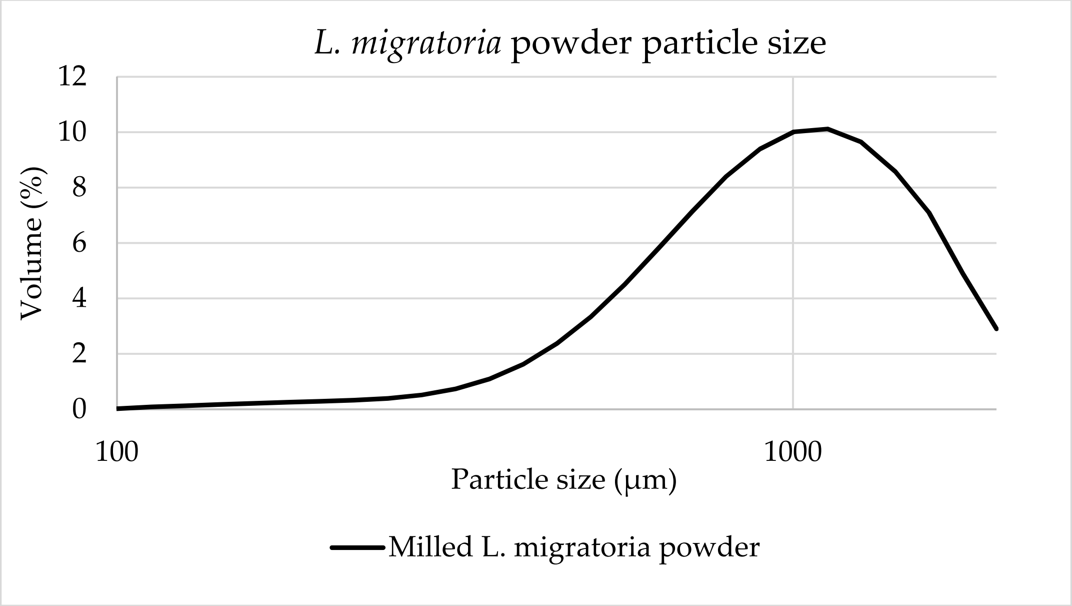

Figure S2. Particle size distribution of milled *L. migratoria* powder.
